# Model Order Reduction: A Comparison between Integer and Non-Integer Order Systems Approaches

**DOI:** 10.3390/e21090876

**Published:** 2019-09-09

**Authors:** Riccardo Caponetto, José Tenreiro Machado, Emanuele Murgano, Maria Gabriella Xibilia

**Affiliations:** 1 Dipartimento di Ingegneria Elettrica Elettronica e Informatica, University of Catania, 95125 Catania, Italy; 2 Institute of Engineering, Polytechnic of Porto, 4200-072 Porto, Portugal; 3 Dipartimento di Ingegneria, University of Messina, 98121 Messina, Italy

**Keywords:** fractional calculus, model order reduction, non-convex optimization

## Abstract

In this paper, classical and non-integer model order reduction methodologies are compared. Non integer order calculus has been used to generalize many classical control strategies. The property of compressing information in modelling systems, distributed in time and space, and the capability of describing long-term memory effects in dynamical systems are two features suggesting also the application of fractional calculus in model order reduction. In the paper, an open loop balanced realization is compared with three approaches based on a non-integer representation of the reduced system. Several case studies are considered and compared. The results confirm the capability of fractional order systems to capture and compress the dynamics of high order systems.

## 1. Introduction

A relevant topic in automatic control is the Model Order Reduction (MOR). The MOR approximates a high-order Linear Time-Invariant (LTI) system with a low-order one, neglecting the less significant state-space variables, decreasing therefore the number of variables and parameters needed for its representation, and simplifying the controller design procedure. Other fields where the use of MOR is highly recommended are related to information compression applications and filters design.

Different strategies are presented in the literature, among which, in the case of asymptotically stable LTI systems, one of the most relevant is based on the open loop balanced realization [1]. Other techniques have been defined using either optimization algorithms such as Genetic Algorithm (GA) [2] and Particle Swarm Optimization [3], or using Artificial Neural Networks [4].

In parallel to these methods, new MOR strategies, see [5], have been investigated applying Fractional Order Calculus (FOC) [6], due to its capability for modelling complex physical phenomena with fractional differential equations. The strategy proposed in [5] looks at the $H_{\infty }$ norm of the error between the original and the approximated transfer function and it is restricted to systems with specific proprieties.

In this paper, a comparison between classical and fractional order MOR methodologies is presented. Different optimization algorithms, evolutionary and gradient based, have been applied for obtaining the fractional order reduced models. Suitable indexes for the approximation error in the frequency and time domains are considered. In particular, the open loop balanced realization is compared with three other procedures that provide fractional order reduced systems.

The paper is organized as follows: in Section 2, the FOC is briefly introduced; in Section 3 the adopted MOR techniques are discussed; in Section 3, comparative examples are given and, finally, in Section 4, conclusions are drawn.

## 2. Some Notes on Fractional Calculus

The long-range temporal or spatial dependence phenomena inherent to fractional order systems present unique peculiarities, not supported by their integer order counterpart that allows for better models for the dynamics of complex processes. In many cases, these properties make fractional order systems more adequate than the standard integer order ones. Fractional (or non integer order) systems can be considered as a generalization of integer order formulation [6,7].

The integro-differential operator ${}_{a}D_{t}^{r}$ with a,t∈R as operation limits and α∈R is defined as follows:

(1) $${}_{a}D_{t}^{\alpha }=\left\{\begin{array}{cc} \frac{d^{\alpha }}{dt^{\alpha }} & :\alpha >0,\\ 1 & :\alpha =0,\\ \int_{a}^{t}{\left(d\tau \right)}^{-\alpha } & :\alpha <0. \end{array}\right.$$

To evaluate a fractional-order derivative or integral of a function, three different definitions are used: for continuous-time domain, these are the Riemann–Liouville (RL) and Caputo (C), while, in the discrete domain, the Grunwald-Letnikov (GL) [6,8].

The RL and C definitions for a time-varying function f(t) are defined, respectively, by:

(2) $${\left\{{}_{a}D_{t}^{\alpha }\right\}}_{RL}f\left(t\right)=\frac{1}{\Gamma (n-\alpha )}\frac{d^{n}}{dt^{n}}\int_{a}^{t}\frac{f(\tau )}{{\left(t-\tau \right)}^{\alpha -n+1}}d\tau$$

and

(3) $${\left\{{}_{a}D_{t}^{\alpha }\right\}}_{C}f\left(t\right)=\frac{1}{\Gamma (n-\alpha )}\int_{a}^{t}\frac{f^{\left(n\right)}\left(\tau \right)}{{\left(t-\tau \right)}^{\alpha -n+1}}d\tau ,$$

where n∈N:n−1<α≤n and Γ(·) is the *Euler Gamma* function [9].

The GL definition instead is given by:

(4) aDtαGLf(t)=limh→0h−α∑j=0[t−ah](−1)jαjf(t−jh),

where [·] evaluates the integer part of its argument.

It is worth noticing that, using the Caputo definition, the initial conditions for fractional-order differential equations are in the same form as its integer-order counterpart, even if some adjustments are necessary [10].

Typical applications of FOC can be found in control [11,12,13,14,15,16,17,18,19,20], chaos [21], Fractional Order Element (FOE) [22,23,24,25,26], Fractional Order Impedance (FOI) characterization [27,28,29], and medical applications [30,31,32]. More recently, fractional calculus is being applied in the study of complex systems. The state of the art can be found in [33], where the broad impact of entropy and information theory-based techniques in complexity, nonlinearity, and fractionality has been shown. A further characterizing property of fractional systems is their capability to model systems with memory as in economy and finance [34,35]. Entropy is also investigated in the framework of the fractional calculus [36]. For example, in [37], a fractional order derivative was applied to the probability distribution function introducing a new perspective in entropy definition. In [38], the Rényi entropy, inspired in the concepts of fractional calculus, was compared with other fractional entropies showing a superior sensitivity to the characteristics exhibited by a distinct data set. Entropy, information theory and fractional calculus tools, have been also recently applied for studying the dynamics of a national soccer league. The complex system state, consisting of the goals scored by the teams, is processed by means of different tools, namely entropy, mutual information and Jensen–Shannon divergence [39].

For a fractional order system, it is possible to define the Laplace transform—see [6]. The most common example of fractional-order system is the fractional-order integrator:

(5) $$F\left(s\right)=\frac{1}{s^{\alpha }},$$

where 0<α<1.

**Figure 1:**
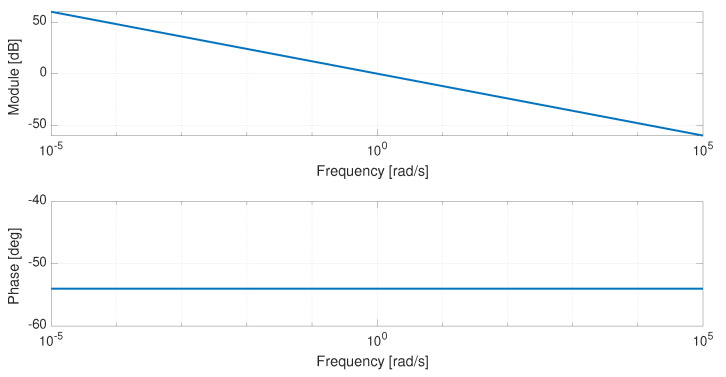
Bode diagram of $F\left(s\right)=100/s^{0.6}$.

As it is possible to observe in Figure 1, the behaviour of the fractional-order integrator is different from that of the integer order one. Evaluating module and phase of Equation (5), it follows that the module is equal to −20αdB/dec, while the phase is constant in all the frequency domain and equal to −απ/2. In the example, with α=0.6, the module has a slope of −12dB/dec, whereas the phase is constant to $-54^{\circ }$. Therefore, selecting only one parameter, it is possible to obtain different dynamics. This property can be useful both in control and in system modelling.

## 3. Description of the Investigated MOR Techniques

In this section, the four different methodologies are presented and briefly discussed.

### 3.1. Open-Loop Balancing Reduction

The open-loop balancing reduction is one of the most relevant techniques used to reduce the model order of a LTI system because it deals directly with the state variables, evaluating the less significant state variables on the basis of their energy contribution. This technique was proposed in [1] and it is valid for asymptotically stable systems.

Given an LTI asymptotically stable system, with state-space matrices $\left\{A,B,C,D\right\}$, completely controllable and observable, it is possible to define two matrices, namely the *controllability* and *observability Gramians*, $W_{c}^{2}$ and $W_{o}^{2}$, respectively. These two matrices are positive definite and symmetrical. The singular values of the controllability Gramian are equal to those of the observability one and their product is a invariant system. The matrices can be evaluated as solutions of the following Lyapunov equations:

(6a) $$\begin{array}{cc} A^{T}P+PA^{T} & =-BB^{T}, \end{array}$$

(6b) $$\begin{array}{cc} A^{T}P+PA^{T} & =-C^{T}C, \end{array}$$

where Equations (6a) and (6b) are the controllability and observability Gramian equations, respectively. The practical computation of Gramian matrices for FOC-LTIs has been recently discussed in [40].

The *system singular values* $\sigma_{1}\geq \sigma_{2}\geq \ldots \geq \sigma_{n}$ are defined as the eigenvalues of the product of the two Gramians and play a fundamental role in the open-loop balancing reduction. In fact, it is possible to determine a particular representation where $W_{c}^{2}=W_{o}^{2}=diag\left(\sigma_{1},\sigma_{2},\ldots ,\sigma_{n}\right),$ that is, where they are coincident and equal to a diagonal matrix, with values that are the square root of the system singular values. This representation is called *Open Loop Balanced Realization* (OLBR) [41] and allows us to analyse both the controllability and observability of the system looking at the same parameters and to select strong and weak observable and controllable state-space variables. Since the system singular values refer to state-space variables energy, if appropriately sorted, they allow neglecting the states whose contributions are less significant. In this way, model order reduction can be performed.

Assuming that the system under investigation is asymptotically stable, completely observable and controllable and represented in the OLBR form, if a singular value $\sigma_{r}$ exists such that $\sigma_{1}\geq \sigma_{2}\geq \ldots \geq \sigma_{r}\gg \sigma_{r+1}\geq \ldots \geq \sigma_{n}$, then two clusters can be defined: the first one that represents the strongest controllable and observable variables, and the second one corresponding to the weakest ones. The system can be decomposed in the following way:

(7) $$\begin{array}{cc} \left[\begin{array}{c} {\dot{x}}_{1}\\ {\dot{x}}_{2} \end{array}\right] & =\left[\begin{array}{cc} A_{11} & A_{12}\\ A_{21} & A_{22} \end{array}\right]\left[\begin{array}{c} x_{1}\\ x_{2} \end{array}\right]+\left[\begin{array}{c} B_{1}\\ B_{2} \end{array}\right]u, \end{array}$$

(8) $$\begin{array}{cc} y & =\left[\begin{array}{cc} C_{1} & C_{2} \end{array}\right]\left[\begin{array}{c} x_{1}\\ x_{2} \end{array}\right], \end{array}$$

where $x_{1}=\left[x_{1}\;x_{2}\;\ldots \;x_{r}\right]$ and $x_{2}=\left[x_{r+1}\;x_{r+2}\;\ldots \;x_{n}\right]$, correspond to the strongest and weakest controllable and observable state-space variables, respectively. If the direct truncation method is applied, then the equivalent reduced system will be the following one, supposing D=0:

(9) $$\left\{\begin{array}{cc} \dot{x_{1}} & =A_{11}x_{1}+B_{1}u,\\ y & =C_{1}x. \end{array}\right.$$

It means that the weakest part is neglected setting $x_{2}=0$.

In the following, this method will be labelled with Method in [1], according to the related reference number.

### 3.2. Implicit Model Order Reduction via Fractional Order Calculus

In this section, the implicit model order reduction introduced in [5] is reported. The method differs from the open-loop balancing reduction because it deals with the system representation via transfer function instead of state-space matrices. The system transfer function must have all poles and zeros real and negative, and assumes the following form:

(10) $$G\left(s\right)=K\frac{\sum_{\begin{array}{c} i=0 \end{array}}^{m}\left(1+\frac{s}{\omega z_{i}}\right)}{\sum_{\begin{array}{c} i=0 \end{array}}^{n}\left(1+\frac{s}{\omega p_{i}}\right)},$$

where $\omega_{z_{i}}$, $\omega_{z_{i}}$ and *K* are the zeros, poles and gain of the system.

The system modelled in Equation (10) is reduced via the following *implicit model*:

(11) $$\tilde{G}\left(s\right)=\frac{K_{reduced}}{{\left(1+\frac{s}{p_{T}}\right)}^{\alpha }},$$

where $p_{T}$ is the transitional frequency and α is the integration order. As a first step of the model reduction, the poles $p_{i}$ and zeros $z_{i}$ are organized into two vectors, sorted in increasing order, respectively $P=\left[p_{1},p_{2},\ldots ,p_{n}\right]$ and $Z=\left[z_{1},z_{2},\ldots ,z_{m}\right]$ and a unique vector $S=\left[Z,P\right]$ is built. Defining the minimum number of parameters $N_{min}$ to be used in the compression, all possible combinations with at least $N_{min}$ elements are extracted from *S*. All of these vectors must have a zero in the even index and a pole in the odd one. Furthermore, the first and the last elements must be poles. The reduced system will be chosen starting from these combination and defining $p_{T}$ and α according to the procedure introduced in [5]. The implicit model approach requires that the system must have the first pole smaller than the first zero; only a pair of complex conjugate poles and the poles and zeroes must be real and negative.

In the following, this method will be labelled with Method [5], according to the related reference number.

### 3.3. Fractional Order Transfer Function Fitting

The third approach starts from the choice of one of the following approximation functions:

(12a) $$\begin{array}{cc} {\tilde{G}}_{1}\left(s\right)= & \frac{k}{{\left(1+\frac{s}{p_{T}}\right)}^{\alpha }}, \end{array}$$

(12b) $$\begin{array}{cc} {\tilde{G}}_{2}\left(s\right)= & \frac{A}{s^{\alpha }+B}, \end{array}$$

(12c) $$\begin{array}{cc} {\tilde{G}}_{3}\left(s\right)= & \frac{s^{\alpha }+A}{s^{\beta }+Bs^{\gamma }+C}, \end{array}$$

(12d) $$\begin{array}{cc} {\tilde{G}}_{4}\left(s\right)= & \frac{k}{{\left(1+\frac{s}{p_{t}}\right)}^{\alpha }}\cdot \frac{1}{s+a} \end{array}$$

where A, B and C are real constants.

Equations (12a) and (12b) can be chosen to obtain a fractional order approximation of an integer order system characterized by a low-pass behaviour. Equation (12c) can model not only low-pass systems, but also systems with more zeros because β or γ can assume negative values. Finally, in Equation (12d), a first integer order term is added if the fractional order term is not sufficient to model the system behaviour.

After choosing the approximating function, two optimization procedures are applied, and compared to determine the coefficients of the approximation. In particular, the Genetic Algorithms [42] and the fminsearch [43] procedures are compared. Numerical stimulations were performed by using routines and procedures given in [44,45].

Given *N* samples of the Interger Order Transfer Function (IOTF), G(s), and having specified the desired frequency window for the approximation, a reduced Fractional Order Transfer Funtion (FOTF), $\tilde{G}\left(s\right)$, is chosen and the following cost function *c*:

(13) $$c=\left|\left|G(s)\right|-\left|\tilde{G}\left(s\right)\right|\right|+\left|∠G\left(s\right)-∠\tilde{G}\left(s\right)\right|$$

is minimized to determine the FOTF parameters.

In Equation (13), the sum of each term is evaluated and then averaged with respect to the number of samples. The optimization based on the GAs will be referred to as a Fractional Order Genetic Algorithm *FO-GA*, while the other one with Fractional Order FMINS *FO-FMINS*. The fminsearch approach, when converging to the minimum, reveals sensitivity of the choice of the initial conditions. In order to overcome this problem in the applied *FO-FMINS*, the initial conditions are chosen as the mean value of the domain interval defined in the GA, while in a further step the *FO-FMINS* algorithm is applied five times defining the initial conditions $x_{0}$ for the next iteration as the identified parameters *x* of the previous iteration, i.e., $x_{0,i+1}=x_{i}$.

The parameters adopted for the GAs optimization are reported in Table 1.

## 4. Numerical Examples

In this section, three different IOTFs taken from literature [2,5,41], see Table 2, are considered, and, for each of them, the aforementioned techniques are applied in the frequency range *f*=$\left[10^{-4},10^{2}\right]$ Hz. The comparison analysis is performed fixing the same number of parameters for all the four different reduced models, minimizing the error given in Equation (13).

### 4.1. System with Transfer Function $G_{1}\left(s\right)$

**Figure 2:**
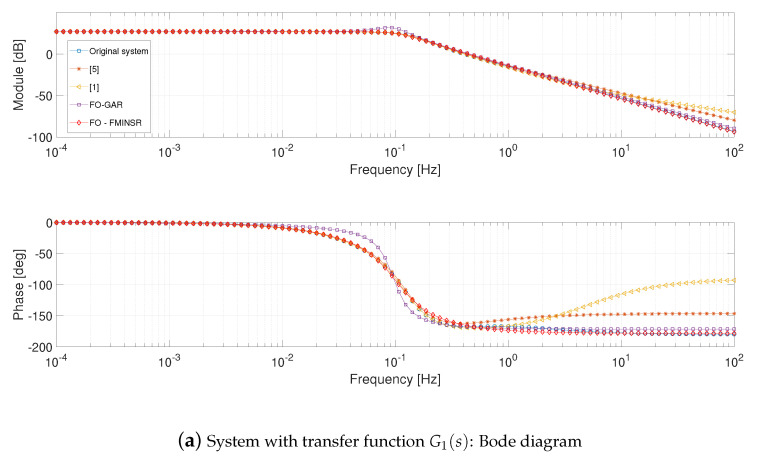

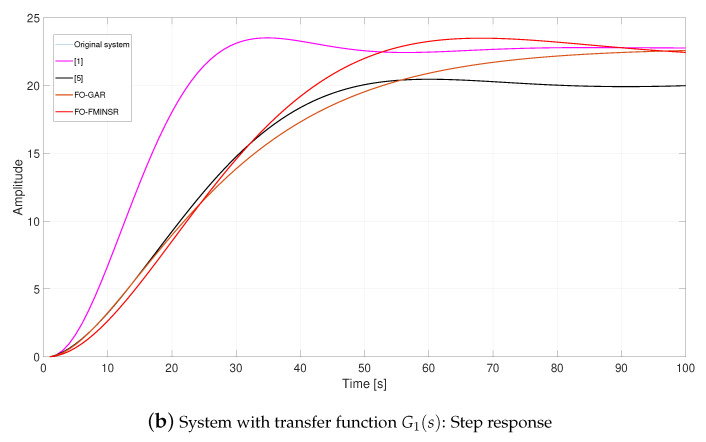
Bode diagram and step response of system with transfer function $G_{1}\left(s\right)$ and corresponding fitted systems.

Considering the $G_{1}\left(s\right)$ system, the frequency responses of the reduced models, see Figure 2a, show a better approximation in the cases of the two methodologies *FO-GA* and *FO-FMINS*. The approximating transfer functions are in the form of Equation (12c) for the *FO-GA* approach and in the form Equation (12d) for the *FO-FMINS*. Independently from the optimization algorithm and from the chosen approximating function, the fractional order models reveal themselves to be quite versatile in the approximation.

If the step responses are considered, see Figure 2b, it is possible to note that the worst performance is given by the implicit method in [5]. The reductions *FO-GA* and *FO-FMINS* provide the same steady state condition, while the best approximation is achieved by applying the balanced realization in [1]. The performance degradation in the step responses of *FO-GA* and *FO-FMINS* is due to the fact that the error function takes into account only the frequency domain responses.

### 4.2. System with Transfer Function $G_{2}\left(s\right)$

**Figure 3:**
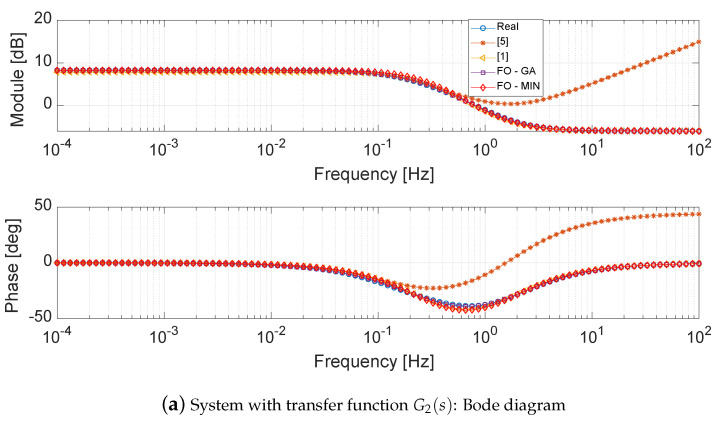

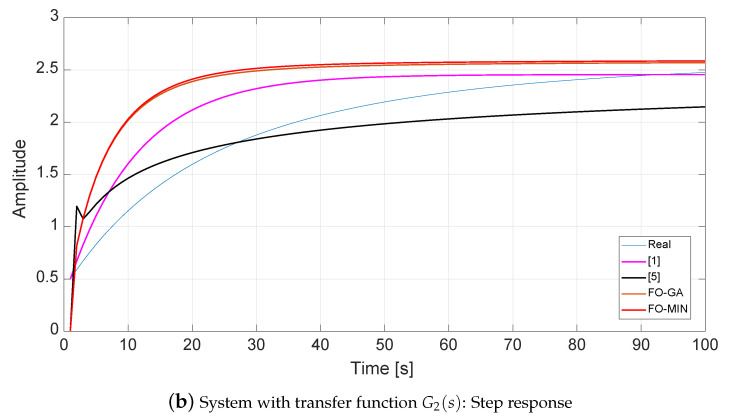
Bode diagram and step response of system with transfer function $G_{2}\left(s\right)$ and corresponding fitted systems.

The transfer function $G_{2}\left(s\right)$ represents an improper system. In the case of the implicit model [5], a zero of $G_{2}\left(s\right)$ could not be taken into account in the reduction. In fact, in the vector *S* (see Section 3.2), the first and last position elements must be poles. Therefore, one zero will remain out of the approximation. The effect of the zero will start from the fourth decade, see Figure 3a, with the visible increase of the module and phase in the frequency approximation. On the contrary, the *FO-GA* approach is able to reduce the original system using a reduced improper transfer function with six parameters. In particular, the two parameters α=0.9529 and β=1.0012 bring to a reduced system in an improper form. The same consideration can be done for the *FO-FMINS* because, with α=0.9378, γ=0.9302 and neglecting β=0.0023, the reduced system is also in an improper form. The best approximation is provided by the balanced approach [1] that provides a simple and effective first order reduced model.

The step responses, see Figure 3b, confirm the previous considerations. In fact, the better approximation is that provided by [5]. The responses of the *FO-GA* and *FO-GA* are very to close each other, confirming the equivalence of the two reduction procedures.

### 4.3. System with Transfer Function $G_{3}\left(s\right)$

**Figure 4:**
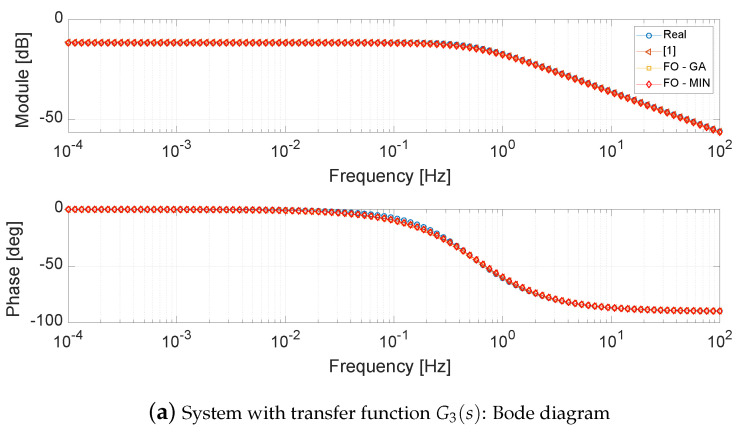

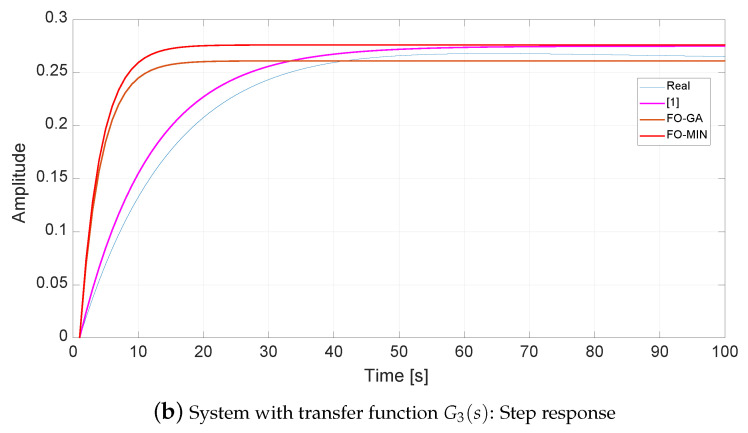
Bode diagram and step response of system with transfer function $G_{3}\left(s\right)$ and corresponding fitted systems.

With respect to system $G_{3}\left(s\right)$, it is possible to note that the implicit method [5] cannot be applied because the system has only one zero whose value is lower than the smaller pole. Looking at the frequency response, see Figure 4a, it is possible to see that the three methods are almost equivalent, guaranteeing the same performances in the frequency domain. On the other hand, by analysing the step responses shown in Figure 4b, it is possible to note that, during the transient, the response of the system was reduced by means of the balancing-based approach is very close to the response of the original system, while, in the steady state, the minimum error is obtained with the *FO-GA* approach.

Table 3 reports the error computed as in Equation (13). The first term represents the error for the module, while the second one the phase error.

Table 4 and Table 5 show the reduced transfer functions obtained with the compared approaches.

## 5. Conclusions

In the previous section, a number of MOR techniques, based on integer and non-integer order models, was evaluated on four systems. Looking at the approximation error values, it can be observed that the techniques based on optimization algorithms are able to fit the IOTFs with good results.

Another important result is that, when dealing with state-space variables, the method [1] works better because the system energy is directly evaluated during the MOR, while this kind of analysis cannot be performed for the fractional order models [10].

In particular, the fminsearch optimization algorithm requires more iterations due to its high dependency on initial conditions, while the GAs do not suffer from this problem if the parameter ranges are well defined. On the contrary, the *FO-R* does not depend on them but on other constraints linked to system structure. The step responses show that the reduced model with fractional-order optimization algorithms achieve good results at the steady state. Further analysis will be performed looking at some system proprieties in order to find, if it exists, a class of transfer functions for which fractional-order based model reduction gives better results than [1]. Another interesting point is the definition of performance indices that also takes into account the time domain behaviour, see [46]. Future research activities will be devoted to the pseudo state space description [10] in fractional reduced order system.

## Figures and Tables

**Table 1:** GA parameters.

Parameter	Value
Number of individuals	3000
Maximum number of generation	150
Generation gap	0.9
Precision	40

**Table 2:** IOTF to be reduced.

System	Transfer Function
$G_{1}\left(s\right)$	$\frac{\begin{array}{cc} (8.51s^{6}+169s^{5}+1279s^{4} & +\\ +4702s^{3}+8834s^{2}+7990s+2675) \end{array}}{\begin{array}{c} (s^{8}+22.52s^{7}+191.1s^{6}+782.9s^{5}+\\ +1684s^{4}+2031s^{3}+1475s^{2}+632.1s+117.6) \end{array}}$
$G_{2}\left(s\right)$	$\frac{\begin{array}{c} 0.5s^{4}+9s^{3}+47.5s^{2}+95s+62 \end{array}}{\begin{array}{c} \left(s+1)(s+2)(s+3)(s+4\right) \end{array}}$
$G_{3}\left(s\right)$	$\frac{\begin{array}{c} s^{3}+11s^{2}+36s+26 \end{array}}{\begin{array}{c} s^{4}+14.6s^{3}+74.96s^{2}+156.7s+99.65 \end{array}}$

**Table 3:** Errors amomg full and reduces order systems.

System	Method in [1]	Method in [5]	FO-GA	FO-FMINS	No. of param.
$G_{1}\left(s\right)$	0.0101+18.2166	0.0579+10.3315	0.4838+3.9158	0.1246+1.7340	[6,6,6,6]
$G_{2}\left(s\right)$	0.069+0.7816	0.6081+15.7925	0.0093+0.2143	0.011+0.2748	[2,4,6,5]
$G_{3}\left(s\right)$	0.0073+0.4806	not applicable	0.0032+0.4730	0.003+0.466	[2,-,2,2]

**Table:** (**a**)

System	Method in [1]
$G_{1}\left(s\right)$	$\frac{0.1983s^{2}+6.243s+6.766}{s^{3}+1.287s^{2}+0.986s+0.2972}$
$G_{2}\left(s\right)$	$\frac{0.5s+4.636}{s+1.888}$
$G_{3}\left(s\right)$	$\frac{1.01}{s+3.674}$

**Table:** (**b**)

System	Method in [5]
$G_{1}\left(s\right)$	$\frac{6.1053}{{\left(1+s/0.4519\right)}^{0.6271}}\cdot \frac{s+2.2387}{s^{2}+0.8s+0.6}$
$G_{2}\left(s\right)$	$\frac{0.2424}{{\left(1+s/0.888\right)}^{0.504}}\cdot \left(s+10.6552\right)$
$G_{3}\left(s\right)$	not appliable

**Table:** (**a**)

System	FO-GA
$G_{1}\left(s\right)$	$\frac{1}{{\left(1+s/0.7252\right)}^{1}}\cdot \frac{17.2505s^{2}+564.9252}{s^{2}+35.3017s+24.825}$
$G_{2}\left(s\right)$	$\frac{s^{0.9529}+9.7293}{s^{1.0012}+1.2876s^{0.84888}+3.7548}$
$G_{3}\left(s\right)$	$\frac{0.2610}{{\left(1+s/3.6157\right)}^{1}}$

**Table:** (**b**)

System	FO-FMINS
$G_{1}\left(s\right)$	$\frac{s^{0.0526}+10.6951}{s^{0.8976}+1.6149s^{1.9872}+0.5007}$
$G_{2}\left(s\right)$	$\frac{s^{0.9378}+8.8892}{s^{0.0023}+2.1085s^{0.9302}+2.426}$
$G_{3}\left(s\right)$	$\frac{0.9570}{{\left(s+3.667\right)}^{1}}$

## Abbreviations

The following abbreviations are used in this manuscript:

MOR	Model Order Reduction
FOC	Fractional Order Calculus
GA	Genetic Algorithm
IOTF	Integer Order Transfer Function
α	Order of Differentiation
FOS	Fractional Order System
FOTF	Fractional Order Transfer Function
OLBR	Open Loop Balancing Realization
